# A mechanistic modelling approach of the host–microbiota interactions to investigate beneficial symbiotic resilience in the human gut

**DOI:** 10.1098/rsif.2023.0756

**Published:** 2024-06-20

**Authors:** Marie Haghebaert, Béatrice Laroche, Lorenzo Sala, Stanislas Mondot, Joël Doré

**Affiliations:** ^1^ University Paris-Saclay, INRAE, MaIAGE, Jouy-en-Josas 78350, France; ^2^ University Paris-Saclay, INRIA, MUSCA, Palaiseau 91120, France; ^3^ Micalis Institute, INRAE, AgroParisTech, University Paris-Saclay, Jouy-en-Josas 78350, France; ^4^ University Paris-Saclay, MGP, INRAE, Jouy-en-Josas 78350, France

**Keywords:** host–microbiota interactions modelling, human gut microbiota, colon flows model, colonic crypt model, PDE–ODE coupling

## Abstract

The health and well-being of a host are deeply influenced by the interactions with its gut microbiota. Contrasted environmental conditions, such as diseases or dietary habits, play a pivotal role in modulating these interactions, impacting microbiota composition and functionality. Such conditions can also lead to transitions from beneficial to detrimental symbiosis, viewed as alternative stable states of the host–microbiota dialogue. This article introduces a novel mathematical model exploring host–microbiota interactions, integrating dynamics of the colonic epithelial crypt, microbial metabolic functions, inflammation sensitivity and colon flows in a transverse section. The model considers metabolic shifts in epithelial cells based on butyrate and hydrogen sulfide concentrations, innate immune pattern recognition receptor activation, microbial oxygen tolerance and the impact of antimicrobial peptides on the microbiota. Using the model, we demonstrated that a high-protein, low-fibre diet exacerbates detrimental interactions and compromises beneficial symbiotic resilience, underscoring a destabilizing effect towards an unhealthy state. Moreover, the proposed model provides essential insights into oxygen levels, fibre and protein breakdown, and basic mechanisms of innate immunity in the colon and offers a crucial understanding of factors influencing the colon environment.

## 1. Introduction

The gut microbiota, an intricate and dynamic ecosystem comprising billions of microorganisms, has become the focus of substantial scientific interest in past and recent years [1]. Indeed, it plays a crucial role in various physiological processes, such as metabolism regulation and immune system functioning. Investigating the interactions between the gut microbiota and host cells enables us to delve deeper into understanding how these microorganisms contribute to human health and diseases [2]. The host shapes bacterial behaviour and composition. Epithelial cells in the colon, responsible for functions like nutrient absorption and immune regulation, control bacterial overgrowth [3]. Epithelial mucus layers act as a barrier and glycoprotein source, influencing microbial colonization and gut microbiota composition [4]. In response, the bacterial ecosystem activates innate immune receptors, impacting cell functions and antimicrobial peptide (AMP) production [5,6].

This intricate symbiosis between the human host and its intestinal microbiota can be either beneficial or detrimental [7]. It has been suggested that shifts in host–microbiota interactions from a beneficial, healthy symbiosis to a detrimental, unhealthy one could be related to ecological transitions between alternative stable states [8–10], associated with environmental factors. However, most of these works, as well as proposed approaches for transition detection in other contexts [11,12], are data-driven, in a context where data collection is complex and costly. Moreover, they lack a mechanistic background.

To fill these gaps, we propose a mathematical modelling approach to investigate the destabilization of a healthy host–microbiota symbiosis.

We focus on a major driver, the host diet, which significantly shapes the gut microbiota, impacting overall health. Western diets, high in protein and low in fibre, are associated with less diverse and beneficial microbiota [13]. Notably, diets low in fibre can cause higher oxygen levels in the gut, primarily owing to the diminished production of short-chain fatty acids (SCFAs), such as butyrate, which are generated through the fermentation of dietary fibres by beneficial gut microbes [14,15]. Butyrate, consumed by colonocytes in an oxygen-dependent manner, plays a critical role in maintaining low oxygen conditions in the gut. A decrease in butyrate production elevates luminal oxygen concentrations, affecting bacterial growth, activity, spatial distribution and epithelial cell function [14,16].

Conversely, diets rich in fibre, such as the Mediterranean diet, support a diverse and beneficial gut microbiota. High fibre intake enhances the production of SCFAs, which serve as an essential energy source for epithelial cells and exhibit anti-inflammatory properties. This contributes to the maintenance of the gut barrier and overall gut health [17–19].

Various models aimed to elucidate facets of the dynamic relationship between the host and its microbiota. Geometric colon models, like [20,21], encompassed microbiota dynamics and environmental conditions. Others, such as [22–24], focused on bacterial communities, providing a basic physical description of the colon. However, none incorporate epithelium dynamics during direct interactions with the microbiota and colon physiological mechanisms. The colonic crypt model by Darrigade [25] captured epithelial cell differentiation, proliferation dynamics and the influence of butyrate and oxygen diffusion within the crypt, linking microbiota presence to butyrate. Researchers exploring innate immunity reactions have developed models investigating receptor activation [26], pathogen invasion, AMP production [27], specific bacteria-induced receptor activation [28] and incorporating adaptive immune system components for allergy development [29].

**Figure 1.**
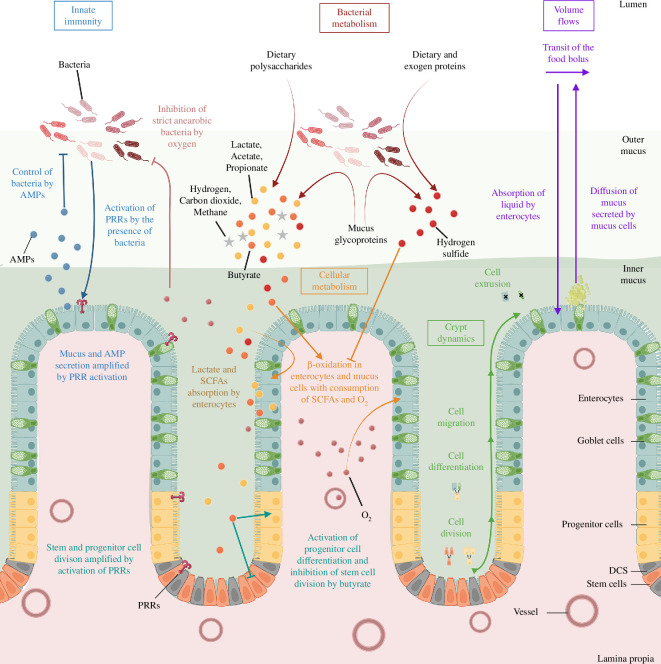
Biological representation of the main symbiotic mechanisms present in our model. This figure delineates the key interactions between the host and its gut microbiota as represented in the model. The major components of the model are enclosed in coloured boxes, with text and arrows in corresponding colours to describe the mechanistic aspects. Bacterial metabolism is highlighted in red. It accounts for dietary fibres and protein as well as host-derived mucus glycoprotein degradation, microbial metabolites production (lactate, SFCAs: acetate, propionate, butyrate) and gases: hydrogen, carbon dioxide, hydrogen sulfide, methane and oxygen. Volume flows, representing transit, mucus secretion and epithelial absorption, are indicated in purple. Innate immunity mechanisms are in blue, they include AMP secretion and control of microbes as well as pattern recognition receptor (PRR) activation. Epithelial cell populations include stem cells, deep secretovolume flows in itry cells (DCS), progenitor cells and differentiated cells (goblet and enterocytes). The metabolism of differentiated cells, focused on the β-oxidation of SCFA, is in orange, and crypt dynamics, comprising mechanical interactions and cell fate events, in green. Note that the graphical elements are not to scale. Created with BioRender.com.

**Figure 2.**
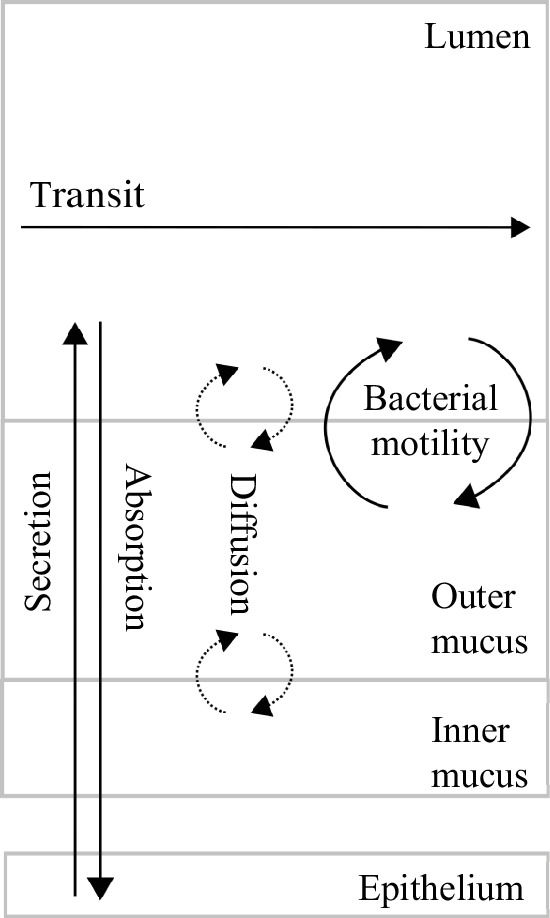
Schematic representation of flows in the model. We represent transit, absorption, secretion, diffusion and bacterial motility flows within the model compartments. The epithelium is depicted by the crypt model. Secretion refers to mucus, AMP and oxygen production; absorption refers to liquid, dissolved metabolites and $H_{2}S$ . Diffusion is linked to gases and monosaccharides, bacterial motility is a shortcut for adherence, residence and shear effects and finally, transit flow influences all state variables in the lumen compartment.

**Figure 3.**
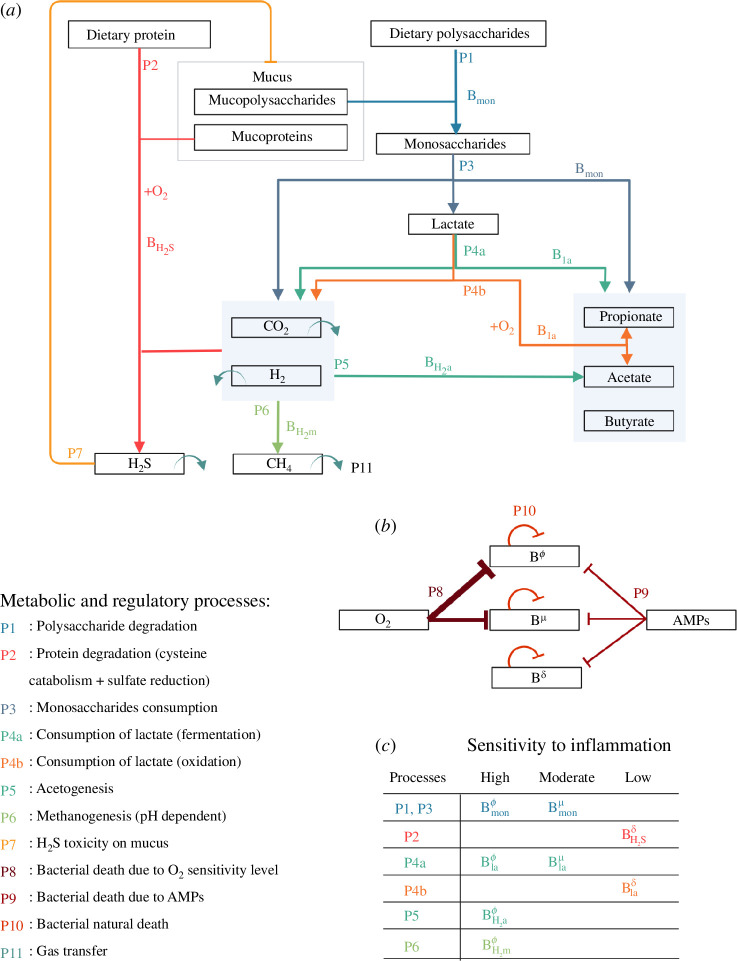
Metabolic and regulatory processes representation. This diagram elucidates the intricate relationships between various microbial groups that participate in the degradation of polysaccharides and protein breakdown as depicted in our model. (*a*) Each metabolic process is designated by a unique number and colour. Arrows originate from the reaction substrates and culminate at the end-products, linking them to the corresponding microbial groups. The term $+O_{2}$ denotes oxygen consumption and signifies an oxidation reaction. Related end-products are grouped within light-blue boxes. We also represent the transfer from liquid to gaseous phase of methane ( $CH_{4}$ ), carbon dioxide ( $CO_{2}$ ), hydrogen ( $H_{2}$ ) and hydrogen sulfide ( $H_{2}S$ ). Regulatory processes included in the model encompass $H_{2}S$ toxicity on mucus bounds. (*b*) The model accounts for basal and AMPs and oxygen concentration-dependent death rates. Here, oxygen is a proxy for inflammation, with three sensitivity levels for the microbes: high ( ϕ ), moderate ( μ ) and low ( δ ). The proposed partition into eight microbial groups is provided in panel (*c*).

Our model, summarized in figure 1, is pioneering, concurrently representing colonic crypt dynamics, mucus, innate epithelial response, fibre and protein degradation by functional bacterial groups and metabolite diffusion in the environment, in order to offer insights into host–microbiota interactions and health impact. The model is placed in a colon section and bridges the gap between crypt and colon scales, it encompasses volume flows between compartments as depicted in figure 2. It results from substantial improvements brought to the crypt dynamics representation in [25], including new metabolite diffusion, absorption and important cellular metabolic switches. Drawing on [21] and [20], the microbiota representation is also inspired by Kettle *et al*. [30]. As shown in figure 3, in addition to fibre input, we complemented [21] with a dietary protein input and the associated microbial metabolic processes. Moreover, based on data analysis (see electronic supplementary material, appendix E), we introduced a representation of microbial groups accounting for their metabolic abilities and sensitivity to inflammation. We included basal innate immunity responses influencing cell division, mucus and AMP production, crucial for symbiosis.

Section 2 introduces the model and its simulation. The ability to simulate a healthy state is demonstrated in §3. The model investigated diet effects by simulating various fibres and protein inputs on symbiosis biomarkers. Assessing the resilience of beneficial interactions or the transition to an unhealthy symbiosis under a high protein (HP)/low fibre (LF) diet was done by mimicking an epithelial barrier breach. Section 4 discusses the hypotheses underlying this work and addresses future perspectives.

## 2. Model and methods

### 2.1. Microbiota model integrating metabolic functions and sensitivity to inflammation

Metabolic functions encompass the biochemical reactions in bacteria and archaea, involving nutrient uptake, growth and waste product excretion. Sensitivity to inflammation indicates bacterial species’ varied reactions in the colon, influencing survival, growth reduction or death. Integrating these aspects into a single model is challenging owing to their interdependence and dynamic nature.

#### 2.1.1. Metabolic process-based microbial groups

Our model includes five metabolic microbial groups responsible for polysaccharide and protein degradation. This group-based approach leverages metabolic redundancy among microbial species for efficiency.

##### 2.1.1.1. Polysaccharide degradation

As described by Muñoz-Tamayo *et al.* [21], the fermentative breakdown of polysaccharides involves multiple bacterial groups interconnected by trophic relationships. The first group, $B_{mon}$ , hydrolyses polysaccharides that originate from both dietary sources and mucus, thereby generating monosaccharides. It metabolizes these for growth, producing lactate, SCFAs (acetate, propionate and butyrate) and releasing hydrogen ( $H_{2}$ ) and carbon dioxide ( $CO_{2}$ ). The second group, $B_{la}$ , uses lactate for growth, producing SCFAs, $H_{2}$ and $CO_{2}$ . Within $B_{la}$ , two pathways are proposed: oxidative and fermentative, with the oxidative pathway involving oxygen consumption. Groups $B_{H_{2}a}$ and $B_{H_{2}m}$ are hydrogenotrophic microorganisms using $H_{2}$ and $CO_{2}$ for energy. They produce acetate and methane ( $CH_{4}$ ), with pH-dependent methanogenesis modelled by the parameter $I_{pH}$ [21]. It should be noted that while our discussion predominantly pertains to bacteria within the microbiota, $B_{H_{2}m}$ specifically denotes methanogenic archaea.

##### 2.1.1.2. Protein breakdown and hydrogen sulfide ( $H_{2}S$ ) production

Building upon the original framework posited by Muñoz-Tamayo *et al.* [21], we sought to refine and extend the bacterial representation within the model. Previously, the model accounted for only two hydrogenotrophic microorganisms (acetogens and methanogens). This work delved into the incorporation of a third category: sulfate-reducing bacteria (SRB).

SRBs are recognized for using hydrogen to synthesize $H_{2}S$ , a critical process in the interaction of host–microbiota. Indeed, in moderate concentrations, epithelial cells metabolize $H_{2}S$ , but when the concentration exceeds a certain limit, it becomes toxic, inhibiting β-oxidation. The latter is a cell metabolic process where SCFAs are decomposed to produce energy while consuming oxygen [31]. Moreover, elevated $H_{2}S$ levels also compromise mucus bonds, undermining the efficacy of the mucus network [31]. While $H_{2}S$ production is attributed to SRB activity, it is also significantly generated through cysteine catabolism [31], a constituent amino acid of proteins.

To address $H_{2}S$ production, we introduced a novel bacterial group within our model, the $B_{H_{2}s}$ group. This group encompasses not only SRB but also those bacteria capable of cysteine catabolism. It spans a broad spectrum of bacteria, from facultative anaerobes like *Escherichia*, *Salmonella*, *Streptococcus* and *Enterobacter*, to strict anaerobes such as *Desulfovibrio* [31]. The integration of this group enabled the model to account for the presence of protein within the colonic environment. While they are less abundant in the dietary content than fibres, owing to their breakdown in the small intestine, proteins can still find their way to the colon, especially under HP diets [32].

Given the metabolic diversity within this group, we postulated that the $B_{H_{2}S}$ group uses proteins, $H_{2}$ , $CO_{2}$ and $O_{2}$ for growth. Since some members of the group use oxidative pathways, which are more energy efficient than fermentation, we considered a higher division rate for this group. Although $B_{H_{2}S}$ can degrade proteins, contributing to their mucolytic properties, we hypothesized that the energy yield from degrading mucoprotein was lower compared to the breakdown of dietary protein, leading to slower growth and reduced $H_{2}S$ production.

To summarize, the model includes five distinct metabolic process-based bacterial groups, each involved in various stages of polysaccharides and protein degradation pathways (figure 3).

### 2.1.2. Microbial groups inflammation sensitivity index

Inflammatory response is the first immune reaction of the host to protect itself from the gut microbiota. Bacteria are not all equally sensitive to inflammation, some can benefit from this state whereas others can be seriously threatened. Our approach was to propose a bacterial representation combining both metabolism abilities and sensitivity to inflammation. By conducting a time-curve analysis of bacterial populations in rats subjected to inflammation-like perturbations, we were able to discern distinct bacterial responses to inflammation and associate them with the metabolic process-based bacterial groups defined earlier (for the data analysis see electronic supplementary material, appendix E), in accordance with the literature [14,33]. We refined our model accordingly by proposing three inflammation sensitivity levels for the functional microbial groups: ϕ for high sensitivity, μ for those demonstrating medium sensitivity, and δ for those displaying tolerance or even promotion by inflammation. Moreover, we chose the oxygen concentration as our primary marker for inflammation. This choice is underpinned by a correlation: increased inflammation often coincides with a surge in oxygen concentration within the colon [14].

Specifically, bacteria that consume monosaccharides have been bifurcated into two distinct subgroups based on oxygen tolerance: $B_{mon}^{\phi }$ and $B_{mon}^{\mu }$ . A similar distinction has been made for lactate fermenters, designated as $B_{la}^{\phi }$ and $B_{la}^{\mu }$ . We previously introduced two bacterial groups exhibiting oxidative metabolism: the lactate consumers and the hydrogen sulfide producers. Intrinsically, these two groups are believed to be more resilient to inflammation, given their metabolic reliance on oxygen and are attributed a low or null sensitivity level: $B_{la}^{\delta }$ and $B_{H_{2}s}^{\delta }$ . On the flip side, the microbial groups $B_{H_{2}a}^{\phi }$ and $B_{H_{2}m}^{\phi }$ , recognized for their oxygen sensitivity, were directly attributed a heightened vulnerability to inflammatory conditions. We used the oxygen level-dependent death rate in the model to represent this sensitivity. In addition, we emphasized microbial groups to undergo natural and AMP-related death rates (figure 3*b,c*). With a non-targeted effect, AMPs produced by epithelial cells are part of the innate immunity allowing bacterial control. Their concentration, higher in the inner mucus, improves the epithelial barrier.

### 2.2. Host-physiology modelling

Existing models, as proposed by [20,21,30], provided a holistic view of the entire colon. In contrast, our intention was to bridge a comprehensive colon model with the more localized model of epithelial crypt presented in [34]. This necessitated addressing the scale discrepancy between a complete depiction of the colon and the microscopic representation of the epithelial crypt.

To accomplish this, we chose to focus on a small section of the transverse colon, which effectively bridges these different spatial scales, facilitating the dynamic interplay we aimed to investigate. This narrow scope enabled us to sidestep the complexity of fluid mechanics, well described in [20], which, while comprehensive, was not particularly relevant to our present exploration.

The transverse colon was chosen as the site of our sectioning for several reasons. It allowed us to avoid modelling issues related to the periodicity of transit specific to the descending colon, which made it an appealing candidate. Moreover, its higher bacterial density rendered it more relevant to our study than the ascending colon.

This selected colon section was modelled using an ordinary differential equation (ODE) compartmental model that is volume-conservative and influenced by both longitudinal and transverse flows. These transverse flows resulted from the coupling with the crypt model, where the absorption and secretion were modulated by epithelial cells (see figure 1). In the following section, we begin by shedding light on the compartmental model and the integration of the comprehensive metabolic and inflammatory response microbial model. We then proceed to present briefly the crypt model and outline the enhancements we propose based on the model developed in [34].

#### 2.2.1. Compartmental model of colon flows

**Figure 4.**
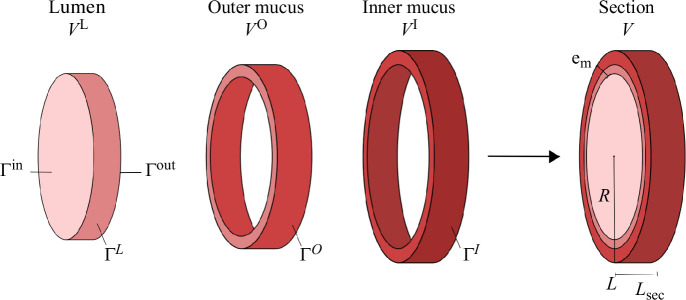
Diagram illustrating the geometrical representation of the compartmental model of the colon section. The model consists of three compartments: lumen, outer mucus and inner mucus, their respective volumes are denoted by $V^{x}\;\forall x\in X$ . The interface between the lumen and outer mucus compartments is denoted by $\Gamma^{L}$ , the interface between the outer mucus and inner mucus compartments is denoted by $\Gamma^{O}$ , and the interface between the inner mucus and the epithelium is denoted by $\Gamma^{I}$ . The total length of the section is $L_{sec}$ , and the radius is R , while the total mucus thickness is $e_{m}$ . The input and output surfaces of the system are represented by $\Gamma^{in}$ and $\Gamma^{out}$ .

The colon flows model is located within a section of the transverse colon, assuming a cylindrical geometry (figure 4). This section is divided into three volume-preserving spatial compartments: the lumen ( L ), the outer mucus phase ( O ) and the inner mucus phase ( I ), gathered in X={L,O,I} (figure 2). Unlike [20,21], we expanded the representation of mucus by differentiating between the outer and inner mucus phases, with the inner phase typically remaining bacteria-free in healthy scenarios. Moreover, we assumed the lack of longitudinal flows near the gut wall owing to the mucus acting as a gel phase.

Following the methodology in [20], we leveraged mixture theory to delineate the evolution of solid phases, particularly focusing on components large enough to exert mechanical forces. We assumed that all phases of the mixture components in the model bear the same density as water and we tracked the volume fraction dynamics within each compartment. Dissolved components are represented as uniformly distributed concentrations in the compartments as in [20].

The solid components include mucus ( m ), polysaccharides ( pol ), proteins ( prot ), dietary residuals ( r ), liquid chyme ( l ) and the eight functional bacterial groups denoted as $B=\{B_{mon}^{\phi },B_{mon}^{\mu },B_{la}^{\phi },B_{la}^{\mu },B_{la}^{\delta },B_{H_{2}s}^{\delta },B_{H_{2}a}^{\phi },B_{H_{2}m}^{\phi }\}$ presented in §2.1. Solid components are compiled in V=B∪{m,pol,prot,r,l} .

Dissolved components are gathered in the set D={mon , la , ac , pro , but , $CH_{4}$ , $CO_{2}$ , $H_{2}$ , $H_{2}S$ , $O_{2}$ , AMPs} , symbolizing monosaccharides ( mon ), lactate ( la ), acetate ( ac ), propionate ( pro ), butyrate ( but ), methane ( $CH_{4}$ ), carbon dioxide ( $CO_{2}$ ), hydrogen ( $H_{2}$ ), hydrogen sulfide ( $H_{2}S$ ), oxygen ( $O_{2}$ ) and AMPs . All gases in the model are denoted by $G=\{\;CH_{4}$ , $CO_{2}$ , $H_{2}$ , $H_{2}S\}$ . Oxygen is omitted in this set as we followed the strategy of [34] to represent it as solute concentration. In accordance with [20], we did not directly incorporate the gaseous phase into our model, instead we assumed gas concentrations to be at equilibrium with the dissolved phase and used a transfer term to represent liquid-to-gaseous phase transformation. This simplification facilitated our focus on the dynamics of solid and liquid phases, refer [21] for the representation of gaseous phase.

The core dynamics of the model are governed by a set of ODEs. These equations dictate the time evolution of the state variables, influenced by the diverse flows occurring in the colon and the presence of bacteria. For each compartment x∈X , the model comprises equations for $f_{i}^{x}$ , representing the mixture phase volume fractions, and $c_{j}^{x}$ , denoting the concentrations of dissolved elements. These equations are expressed for all t>0 as


(2.1) $$\dot{f_{i}^{x}}(t)=\sum_{h\in H}U_{h}^{x}[f_{i}^{x}(t)]+F_{i}^{x}(t)\;\text{and}\;\dot{c_{j}^{x}}(t)=\sum_{h\in H}U_{h}^{x}[c_{j}^{x}(t)]+C_{j}^{x}(t).$$

Here, H={T,S,A,D,M} represents the set of modelled flows: transit ( T ), secretion ( S ), absorption ( A ), diffusion ( D ) and bacterial motion ( M ). Functions $U_{h}^{x}$ , for h∈H , define the form of each of these flows in compartment x . $F_{i}^{x}$ and $C_{j}^{x}$ represent metabolic transformations applied to the mixture phase and dissolved elements, respectively.

As a result of the section representation, we only set transit flow as longitudinal input and output flows $v^{in}(t)$ and $v^{out}(t)$ , within the lumen compartment surfaces $\Gamma^{in}$ and $\Gamma^{out}$ . The transit flow acts on all volume fractions and dissolved components. Because of their volume, polysaccharides ( pol ), proteins ( prot ) and chyme residual ( r ) cannot enter the mucus; therefore, they are only present in the lumen compartment [20].

Secretion flows include epithelial crypt-induced flows occurring from the epithelium to the lumen, they are applied to mucus, AMPs and oxygen. In the inner mucus compartment, mucus and AMP intake flows depend on the total amount of goblet and enterocyte cells present in the section (total number of cells per crypt × number of crypts in the section). We modelled a constant production rate linked to basal cell functioning. With the presence of innate immunity receptors on and in cells: the pattern recognition receptor (PRRs), cells are able to detect bacteria and respond to their presence by producing more mucus and AMPs [3]. Therefore, in addition to basal production, we added a bacteria-dependent production term based on total bacteria density in the outer mucus compartment denoted by $f_{B}^{O}=\sum_{b\in B}f_{b}^{O}$ . We emphasize that mucus becomes liquid when it enters the lumen compartment, representing the disassembly of the mucus network.

Standing in contrast to secretion flows are absorption flows directed from the lumen compartment to the inner mucus compartment. These are instigated by enterocytes located within crypts and apply to both metabolites and hydrogen sulfide ( $H_{2}S$ ), which serve as energy sources for cells [31]. Absorption flows also apply to liquid l , playing a crucial role in food bolus solidification. We highlight that as the wall is approached, absorption rates intensify.

Some state variables, particularly gas and monosaccharide concentrations, do not experience absorption or secretion flows. We assumed that they follow a passive diffusion flow across all compartments.

As in [21], we included bacterial motion in our model as these organisms exhibit both active and passive movements. Passive movements are related to transit and mucus secretion, leading bacteria to follow the longitudinal flow and be vertically driven by mucus production. Moreover, only bacteria exhibit a transit resistance capacity, we used the same expression as in [21] to address this ability. In the model, we highlight that all functional groups of bacteria are attracted to the mucus compartment causing active movement of bacteria from the lumen compartment to the outer mucus compartment. Given that the inner mucus phase is generally bacteria-free or contains a limited number of adaptable bacteria in healthy cases, we modelled that functional groups cannot enter the inner mucus compartment.

In our modelling approach, we maintained constant volumes in compartments. Accordingly, we adjusted the transit flow on $\Gamma^{out}:\;v^{out}(t)$ , the mucus flow between $\Gamma^{I}$ and $\Gamma^{O}:\;v_{m}^{I,O}(t)$ and the mucus flow between $\Gamma^{O}$ and $\Gamma^{L}:\;v_{m}^{O,L}(t)$ at each time t . We should note that the transit slowdown is mimicked by ensuring $0<v^{out}(t)<v^{in}$ . These adjustments were made in response to all volume fraction movements, adhering to the volume conservation principle


(2.2) $$\sum_{i\in V}\dot{f_{i}^{x}}(t)=0,\;t>0,\;x\in X,$$

and considering the mixture theory of volume fraction condition


(2.3) $$\sum_{i\in V}f_{i}^{x}(t)=1,\;t>0,\;x\in X.$$

The specific equations for various flows ( $U_{h}$ for each h∈H ) are in electronic supplementary material, appendix A1. Electronic supplementary material, appendix A3 details the calculations for $v^{out}(t)$ , $v_{m}^{I,O}(t)$ and $v_{m}^{O,L}(t)$ to ensure constant volume compartments.

Finally, the modelling of the metabolic transformations $F_{i}^{x}$ and $C_{j}^{x}$ followed closely [20]. We imposed volume conservation during metabolic processes for the mixture phase components:


(2.4) $$\sum_{i\in V}F_{i}^{x}(t)=0,\;\;t>0,\;\;x\in X.$$

In maintaining a constant volume, our model stipulates that the breakdown of the mixture phase, be it bacterial death or mucus degradation, liberates a corresponding volume of liquid. Similarly, we proposed that bacterial growth is constrained by the available space in the liquid phase and that an equivalent volume of liquid is used during the growth process.

We note $P_{f}^{x}$ (resp. $P_{c}^{x}$ ) the Petersen reaction matrix for compartment x∈X . These matrices define component yields in each of the processes included in the model (figure 3). For each compartment, we also introduced kinetic rate vectors $K_{f}^{x}$ and $K_{c}^{x}$ and set $F^{x}=(F_{i}^{x}{)}_{i\in V}$ and $C^{x}=(C_{j}^{x}{)}_{j\in D}$ as


(2.5) $$F^{x}=P_{f}^{x}.K_{f}^{x}\;\text{and}\;C^{x}=P_{c}^{x}.K_{c}^{x}\;\forall x\in X.$$

$F^{x}$ and $C^{x}$ encompass all metabolic processes of polysaccharides and protein degradation as well as bacterial death mechanisms and hydrogen sulfide toxicity on mucus bounds. Processes labelled P1,…,P11 in figure 3 are elaborated in electronic supplementary material, appendix A2. The definitions of $P_{f}^{x}$ , $K_{f}^{x}$ , $P_{c}^{x}$ and $K_{c}^{x}$ are found in electronic supplementary material, tables A2 and A3.

#### 2.2.2. Space-structured epithelial crypt model

The crypt model, first introduced in [34], represents a deterministic limit of a birth and death process within a piecewise deterministic Markov process, structured by both space and cell type. Distinct from other components of the multi-component model, this specific part uniquely adopts a one-dimensional spatial structure, which directly corresponds to the vertical dimension, or height, within the crypt. The model consists of two interacting parts: cell dynamics evolution within the crypt and solute diffusion, both following partial differential equations (PDEs).

The model traces the evolution of distinct cell types: stem cells ( sc ), progenitor cells ( pc ), goblet cells ( gc ) and enterocytes ( ent ), all modelled as space-structured cell densities. Additionally, it includes the static cell density of deep crypt secretory cells ( dcs ). These cells reside in the crypt and exert mechanical forces upon one another. For concentrations, the model represents the diffusion of oxygen from the bottom of the crypt, originating from vessels beneath the epithelium, to the top leading to its diffusion in the inner mucus compartment, and conversely, the diffusion of butyrate produced by the microbiota from the top of the crypt to the bottom.

With respect to [34], first we adapted the model to represent a human crypt as the original version was based on rodents. Second, we introduced PPRs, already mentioned in the colon flows model description. PPRs are innate immunity receptors present in or on cells that play a key role in host–microbiota symbiosis by acting on cell regulation processes, they impact stem and progenitor cell division [3]. To account for this, in the model, the division process is now positively stimulated by the volume fraction of bacteria in the outer mucus compartment $f_{B}^{O}$ that activate PRRs. We also included lactate, acetate and propionate diffusion along the crypt. We used the inner mucus compartment concentrations $c_{la}^{I}(t)$ , $c_{ac}^{I}(t)$ , $c_{pro}^{I}(t)$ , $c_{but}^{I}(t)$ as boundary conditions for the diffusion PDEs. Furthermore, our model now includes an absorption term for all metabolites, in addition to the β -oxidation term already defined in [34]. Lastly, we modelled a metabolic switch in differentiated cells, which is activated by the level of butyrate and inhibited by the level of hydrogen sulfide ( $H_{2}S$ ) [31,35].

The detailed equations of the crypt model, including the dcs cells density shape, are fully described in electronic supplementary material, appendix B2.

### 2.3. Model simulation

To reconcile differing scales of longitudinal and transverse flow in the colon and visualize the effects of changes, like dietary alterations, we propose simulating a sequence of five colon sections. This sectional approach provides a minimalist spatial representation capturing essential dynamics while ensuring computational efficiency and manageability. The simulation unfolds sequentially, starting with setting inputs for the first section. Inputs for subsequent sections are determined by concentrations and volume fractions obtained at the stationary state of the previous section, ensuring a smooth transition and model consistency. To maintain volume constancy across sections, we adjusted the input flow $v^{in}$ for each section to match the output flow $v^{out}$ of its preceding section at a stationary state. This strategy allowed for more effective visualization of various changes than a single section simulation, yet remained more manageable than a full two-dimensional model.

While it is possible to conduct a simulation of all sections in a stepwise temporal manner, such a procedure can significantly increase the computational cost. Since our primary focus was understanding stationary states rather than transition dynamics, we chose not to employ this comprehensive temporal framework in our primary simulations, though it is feasible and incorporated in the Python source code, available at https://gitlab.com/marie.haghebaert/hostmicrobiota-interactions. This decision balanced simulation accuracy, computational efficiency, and alignment with our objectives. For the numerical methods used in solving the model, see electronic supplementary material, appendix C. Lastly, all parameters used for simulations are compiled in the tables of electronic supplementary material, appendix F.

## 3. Results

Model simulations aim to effectively mimic a healthy human environment. This encompasses elements such as microbial diversity and density, colon physiology and crypt dynamics, under the influence of reference dietary fibre and protein intakes. We leveraged the model’s exploratory capacities to carry out a numerical experiment that illustrates the impact of diet on the model’s stability. This is achieved by studying the effects of variations in protein and fibre intake on bio-markers indicative of symbiosis, such as oxygen concentration in the lumen, PRRs activation rate or the ratio of $B_{H_{2}s}^{\delta }$ bacterial groups. Furthermore, we focused on a specific scenario involving an epithelial barrier breach, a condition that can potentially instigate unhealthy host–microbiota interactions. We examined the differential impacts under a reference healthy diet and a HP/LF diet, which illuminates the model’s capacity to investigate dietary response and homeostasis maintenance.

### 3.1. The reference healthy state

#### 3.1.1. Microbial composition

**Figure 5.**
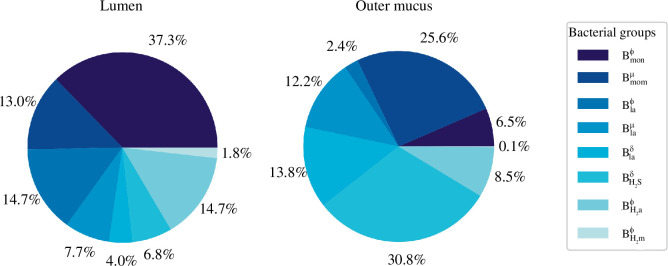
Bacterial group proportions in the lumen and the outer mucus layer. We used plot pie to represent microbiota composition in the lumen and the outer mucus compartment of the model. Results were obtained at a steady state for the last of the five sections analysed.

In our model, we simulated the microbial distribution in the lumen as depicted in figure 5. This closely aligns with the results presented in [20] where monosaccharides degraders, represented as $B_{mon}^{\phi }$ and $B_{mon}^{\mu }$ , are the most prevalent groups (≃ 50%). They are followed by a smaller proportion of lactate consumers (≃ 26%), represented by the $B_{la}^{\phi }$ , $B_{la}^{\mu }$ and $B_{la}^{\delta }$ groups. The hydrogenotrophic groups, $B_{H_{2}a}^{\phi }$ and $B_{H_{2}m}^{\phi }$ , are even less prevalent, respectively, 14.7% and 1.8%. It is noteworthy that bacterial groups sensitive to inflammation (denoted as ϕ ) are found in higher concentrations within the lumen. In contrast, the outer mucus compartment primarily contains bacteria that are either tolerant to inflammation (denoted as μ ) or are insensitive to it (denoted as δ ). Given that our model uses oxygen concentration as an inflammation marker, this distribution aligns well with the anticipated behaviours of strict anaerobes and facultative anaerobes. Specifically, the presence of $B_{la}^{\delta }$ and $B_{H_{2}s}^{\delta }$ at 13.8% and 30.8%, respectively, in this compartment facilitates a decrease in the oxygen concentration within the lumen, owing to their utilization of oxygen during oxidative metabolism. Coupled with β -oxidation, this process aids in maintaining the hypoxic environment of the lumen. Moreover, the modest concentration ( 0.1% ) of $B_{H_{2}m}^{\phi }$ group in the outer mucus compartment reflects the high oxygen sensitivity inherent to methanogenic archaea.

Our analysis revealed a greater bacterial volume fraction within the mucus ( $f_{B}^{O}=0.013$ ), compared to the lumen ( $f_{B}^{L}=0.006$ ). This difference is consistent with the result illustrated in fig. 4 of [20]. Moreover [20] projected the total bacterial volume fraction at the colon’s exit to be approximately 0.06. Given the distal part of the colon is known to harbour a denser bacterial concentration than the transversal one, our results appear to be appropriately scaled.

The bacterial volume in the fifth section, in both the outer mucus and lumen compartments, computed to $f_{B}^{O}\times V^{O}+f_{B}^{L}\times V^{L}=0.125\;cm^{3}$ . To simplify, we posited that all phases possess the same density as water, namely 1 g cm^−3^. Therefore, we estimated the section to accommodate approximately 0.125 g of bacteria, which is a reasonable amount for the transverse colon [20].

#### 3.1.2. Colon physiology

**Figure 6.**
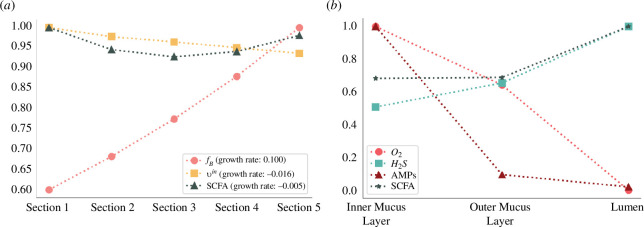
Longitudinal and transverse gradients in colon physiology. (*a*) Evolution of total bacterial fraction volume, total SCFAs concentration and transit flows in the lumen compartment along five sections. Depicted values are divided by their maximum, for SCFAs: 94.5mM , for transit $v^{in}$ : 0.416 cm h^−1^ and for bacterial fraction volume $f_{B}$ : 6.1 × 10^-3^[.]. (*b*) Concentration gradient from lumen to the inner mucus layer for oxygen, $H_{2}S$ , AMPs and SCFAs. Values are divided by their max, for SCFAs: 92.7mM , for $H_{2}S$ : 0.2 mM, for AMPs: 0.47mM and for oxygen 6.7 arb. units.

Concerning longitudinal flows (figure 6*a*), proportionately scaled for this compact representation of the colon, the model faithfully captured the natural increase in bacteria count, reflecting the proliferation of the bacterial load when moving towards the distal colon. The model also correctly depicted the decrease in transit flow, a feature associated with water absorption and stool formation.

In the simulation, we observed a subtle reduction in the total SCFA concentration along the section, as illustrated in figure 6*a*. This aligns with the observed decrease in SCFA concentration throughout the colon [36]. However, the model predicted a concentration of 92.7mM , which is somewhat lower than the biological measurements reported by Bravo & Axelrod [36]. Their study has revealed that the concentration for six individuals averaged approximately 117±9mM in the transverse colon. Moreover, the ratio of SCFAs computed from the model simulation in the lumen for acetate, propionate and butyrate is 54 : 23 : 23, which is close to that observed in [36] of 57 : 21 : 22 in the descending colon.

Turning our attention to the transverse gradients (figure 6*b*), the model successfully generated a hypoxic environment in the lumen, mirroring the natural low-oxygen conditions typical in the colon. This reinforces the model’s accuracy in simulating the colon’s physiological state. In the inner mucus layer, the simulation generated higher concentrations of AMPs compared to the outer mucus layer and the lumen compartments, underlining the protective role of the mucus in preventing bacterial invasion.

$H_{2}S$ concentration decreased from the lumen compartment to the inner mucus one and remained in healthy concentration level <1mM [31].

#### 3.1.3. Epithelial crypt dynamic

##### 3.1.3.1. Epithelial cells

**Figure 7.**
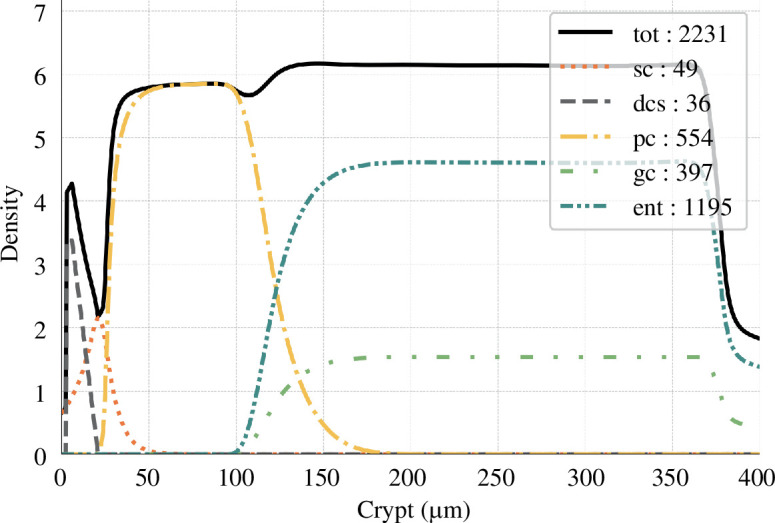
Cell densities along the crypt. Cell densities are plotted as a function of the height in the crypt. The total number of cells is reported in the box for each cell type (sc, stem cells; dcs, deep crypt secretory cells; pc, progenitor cells; ent, enterocytes; gc, goblet cells).

In our simulation (figure 7), the total cell count per crypt is 2231, closely aligning with the literature value of 2427.8 ± 504.4 from [37]. Our model yielded 603 proliferative cells, encompassing both stem and progenitor cells, compared to 623.9 ± 234.1 and 1592 differentiated cells versus 1768.2 ± 434.5 in the cited source. Moreover, the model appropriately replicated the stem cell niche, the proliferative zone, and the differentiated zone. The ratio of goblet cells to enterocytes was observed to be one-third, aligning well with the expected distribution [34].

##### 3.1.3.2. Metabolites and oxygen

**Figure 8.**
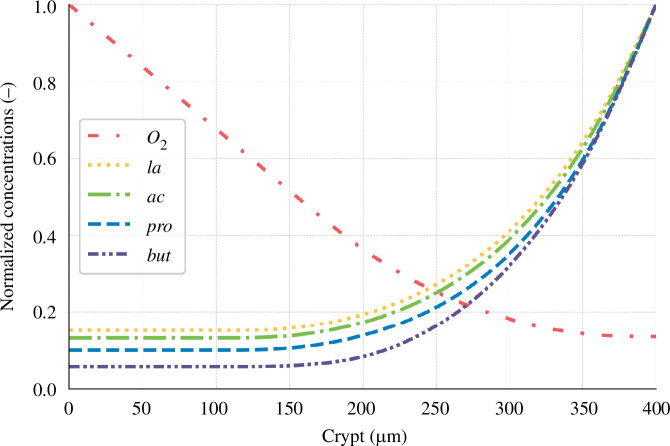
Normalized solute concentration along the crypt. Concentration is divided by their maximum values, for oxygen: 48.8 arb. units, lactate (*la*) : 0.48 mM, acetate (*ac*) : 34.05 mM, propionate (*pro*) : 14.30 mM and butyrate (*but*) : 14.72 mM.

Upon comparison of our simulation results with the existing literature (see figure 8), we found good agreement. For instance, our simulated oxygen gradient from the bottom to the top of the crypt was 7.35. This value is comparable to the findings presented in fig. 1*a* from [14], which depicts the oxygen partial pressure gradient between host tissues and the lumen in a healthy situation.

Our simulations suggest that at the crypt base, SCFA ratios are 66 : 21 : 12. This is in close agreement with the literature-reported ratio of 71 : 21 : 8 observed in the portal vein [36]. Specifically, our model yielded concentrations of 0.83mM for butyrate which corresponds with the upper limit of the reported range of 0.05–0.8 mM [19]. Using the butyrate concentration and the portal vein ratio, we calculated the concentrations of acetate and propionate at the crypt base to fall between 0.44–7.10mM and 0.13–2.1mM, respectively. These calculations align closely with our experimental results, which showed concentrations of 4.46mM for acetate and 1.42mM for propionate.

### 3.2. Numerical exploration to assess diet effect

Building on the validity of our model in reproducing a healthy state, our next objective was to explore its behaviour in response to dietary variations. This aspect of our research is motivated by two primary goals: first, to elucidate the effects of fibre and protein inputs on symbiosis biomarkers, and second, to evaluate the model’s capacity to capture the responses to a HP and LF diet.

#### 3.2.1. Numerical framework

Our experimental design draws from the study of Russel *et al*. [32], which investigated weight-loss diets and provided experimental results for protein and fibre intakes across three diet types: (i) a reference diet with standard protein and fibre intakes, (ii) a HP/high-fibre diet, and (iii) a HP/LF diet. Using results from this research, we derived a realistic range for protein and fibre fraction volume intakes, specifically focusing on the transverse colon to perform an *in silico* experiment.

The volume fraction of polysaccharides introduced at the lumen input surface, $\Gamma^{in}$ , of the initial section was set to lie between 0.02 and 0.06 . Depending on this value, we adjusted the concentrations of various substances: monosaccharide, lactate, acetate, propionate and butyrate, proportionally. Drawing from the values in [32], the concentration ranges were set as follows: $[1\times 10^{-5},4\times 10^{-5}]\;mM$ for monosaccharide, $[2.5\times 10^{-6},5.5\times 10^{-6}]\;mM$ for lactate, $[3\times 10^{-5},7\times 10^{-5}]\;mM$ for acetate, $[1\times 10^{-5},2.5\times 10^{-5}]\;mM$ for propionate, and $[5\times 10^{-6},2.5\times 10^{-5}]\;mM$ for butyrate. The protein volume fraction spanned from 0.01 to 0.03 .

To visualize the data, we used an 8×8 grid in the protein/fibre space and used interpolation for heat map generation. For each parameter space point, we calculated the model across five sections, adjusting the dietary input in the primary section. We then emphasized the steady-state results observed in the fifth section, with each section computed sequentially until $T_{max}=400\;h$ . In our numerical analysis, we prioritized four essential biomarkers serving as indicators of symbiosis:

1. Oxygen concentration in the lumen compartment: an elevated oxygen level promotes a dysbiotic microbiota, fostering facultative anaerobes and inflammation-resistant bacteria.
2. The ratio of $B_{H_{2}s}^{\delta }$ volume to the total bacterial volume in the lumen compartment: an increased proportion of this facultative, inflammation-resistant bacterial group can be seen as a marker of unhealthy host–microbiota interactions.
3. PRRs activation: this marker correlates with the production of AMPs and mucus, vital for sustaining the epithelial barrier.
4. The aggregate count of differentiated cells in the crypt, denoting crypt maturity: crucial to maintain the colon’s healthy functionality in absorption and secretion and ensure a hypoxic lumen environment.

#### 3.2.2. Impact of diet on symbiosis biomarkers

##### 3.2.2.1. Oxygen concentration in the lumen

**Figure 9.**
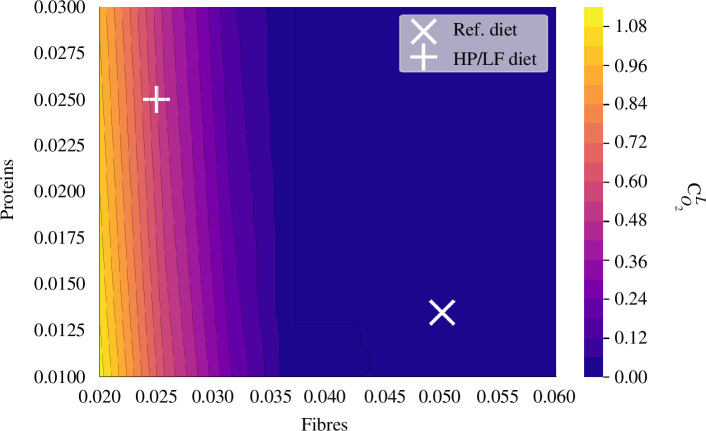
Oxygen concentration in lumen ( $c_{O_{2}}^{L}$ ) influenced by diet. This impact is displayed when varying fibre and protein intake on a grid [0.02,0.06]×[0.01,0.03] as a function of fibre and protein supply.

The concentration of oxygen in the lumen is primarily influenced by dietary fibre input (figure 9). Above a threshold, the oxygen concentration in the lumen approaches 0. However, for LF input, the oxygen concentration increases, mainly owing to the reduced production of butyrate. This, in turn, leads to a lower number of differentiated cells and a potential shift in epithelial cell metabolism, which leaves oxygen coming from vessels beneath the epithelium unconsumed.

##### 3.2.2.2. $B_{H_{2}s}^{\delta }$ group proportion in the lumen

**Figure 10.**
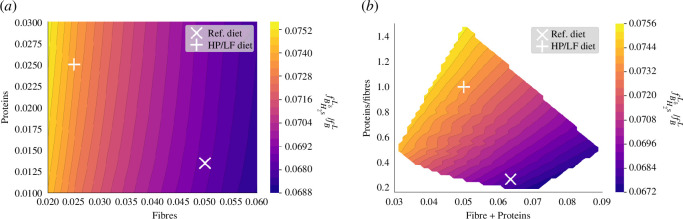
Ratio of $B_{H_{2}s}^{\delta }$ volume to total bacterial volume in the lumen influenced by diet. This impact is displayed when varying fibre and protein intake on a grid [0.02,0.06]×[0.01,0.03] . (*a*) Function of fibre and protein supply. (*b*) Function of the total nutrient supply and the ratio of protein over fibre.

Our model uncovers a correlation with protein intake, suggesting that a HP diet could foster the growth and predominance of this bacterial group (figure 10*a*).

However, an intriguing observation is the mitigating effect of a high-fibre diet against the negative impacts of HP intake. The model illustrates a negative correlation between fibre intake and the proportion of $B_{H_{2}s}^{\delta }$ group, underscoring the role of dietary fibre in nurturing a diverse and balanced gut microbiota. This simulation result suggests that a diet rich in fibre could potentially offset the microbiota dysbiosis induced by a protein-dense diet, echoing findings from [32].

A less intense but significant correlation is also detected between the total dietary intake volume and these bacteria’s proportions, with a decline in facultative anaerobes observed as total intake decreases (figure 10*b*).

##### 3.2.2.3. Pattern recognition receptor activation

**Figure 11.**
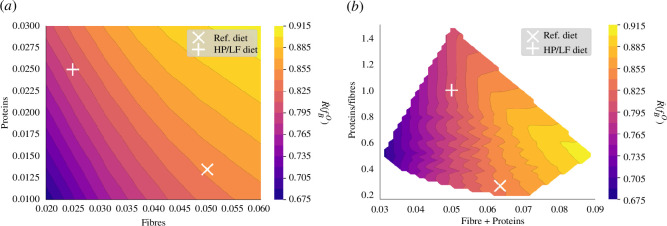
PRRs activation ( $\bar{R}(f_{B}^{O})$ ) influenced by diet. Visualization of the effect of varying fibre and protein supply on PPR activation. This impact is displayed when varying fibre and protein intake on a grid [0.02,0.06]×[0.01,0.03] . (*a*) Function of fibre and protein supply. (*b*) Function of the total nutrient supply and the ratio of protein over fibre.

Our model highlights a positive correlation between PRR activation and the total volume of dietary intake (figure 11*b*). Primarily, this relationship is driven by the decrease in bacterial volume when the diet lacks sufficient fibre and protein content, leading to a simplified carbohydrate degradation trophic chain.

Moreover, we identified a significant secondary correlation between PRR activation and protein intake (figure 11*a*). The facultative anaerobes, specifically the $B_{H_{2}s}^{\delta }$ group, thrive in protein-rich environments and demonstrate increased division and proliferation rates owing to their oxidative metabolism which results in a higher bacterial fraction volume in the mucus compartment.

##### 3.2.2.4. Differentiated cell density within the crypt

**Figure 12.**
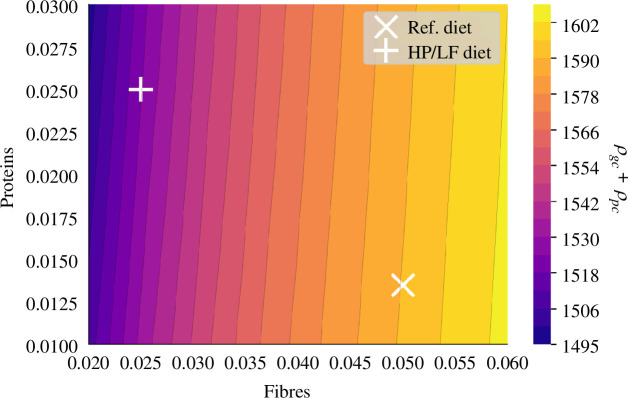
Total number of differentiated cells in one crypt ( $\rho_{gc}+\rho_{pc}$ ) influenced by diet. This number is displayed when varying fibre and protein intake on a grid [0.02,0.06]×[0.01,0.03] as a function of fibre and protein supply.

Dietary intake demonstrably influences crypt maturity (figure 12). A highlight of our study is the positive correlation between crypt maturation and fibre intake. This correlation can be attributed to the role of butyrate in promoting cell differentiation, emphasizing the significance of dietary fibre in the process of crypt maturation. On the other hand, our simulations suggest that protein intake does not have a pronounced impact on crypt maturity in our model.

For a more detailed insight into the effects of protein and fibre on crypts, refer to section 5 in figure 1*3a,c*, which illustrates cell distributions under reference and HP/LF diets. The latter resulted in comparatively immature crypts, characterized by fewer differentiated cells (1514 versus 2190) and more proliferative cells (678 versus 610). Although the numerical differences might appear subtle, the qualitative observations indicate a trend towards crypt immaturity with reduced fibre intake.

**Figure 13.**
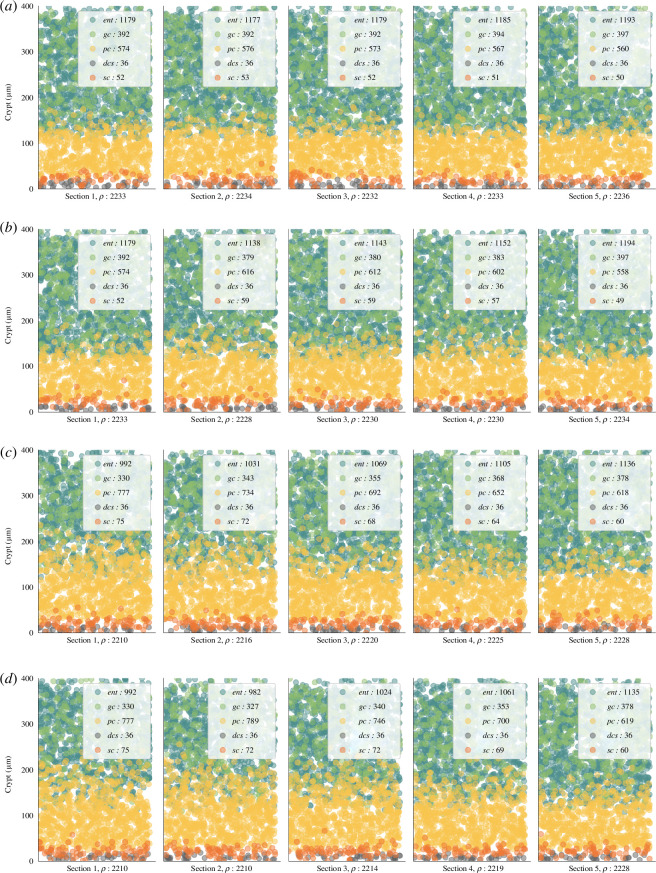
Spatial distribution of cells in crypts under varying diet and breach scenario C. The distribution is visualized in colon sections under different conditions: (*a*) reference diet, (*b*) reference diet with an epithelial breach, (*c*) HP/LF diet, and (*d*) HP/LF diet with an epithelial breach. In each depiction, cells are represented by circles, with their colours indicating distinct cell types. The distribution patterns are based on cell densities derived from the model.

### 3.3. Beneficial symbiotic resilience when facing inflammation episodes

This section is devoted to investigating the impact of diet on the healthy host–microbiota symbiosis stability and resilience. More precisely, we investigated the system’s response to identical inflammatory perturbations under contrasted diet conditions.

#### 3.3.1. Modelling a breach in the epithelial barrier

In this exploration, we replicated a disruption in the epithelial barrier, modelled as an oxygen bloom at the crypt’s bottom and an increase in the AMP production rate symbolizing a deterioration in mucus quality and the corresponding immune system compensation. This disturbance was locally incorporated into colon sections 2–4. See electronic supplementary material, appendix D5 for the parameters used to simulate these scenarios.

#### 3.3.2. Assess the impact of two different diets

To scrutinize the diet’s impact on the resilience of beneficial symbiosis, we contrasted the state in section 5 under two distinct dietary regimes: the reference healthy diet with high fibre and normal protein intake, and a HP/LF diet, highlighted with + and × marks, respectively in figures 9 –12.

The HP/LF diet was modelled with a volume fraction of fibre entering the first section $f_{pol}^{in}=0.025$ and a protein fraction volume entering $f_{prot}^{in}=0.025$ . For this diet, we adjusted monosaccharide, lactate and SCFAs inputs to $c_{mon}^{in}=1.25\times 10^{-5}\;mM$ , $c_{la}^{in}=3.125\times 10^{-6}\;mM$ , $c_{ac}^{in}=3.75\times 10^{-5}\;mM$ , $c_{pro}^{in}=1.25\times 10^{-5}\;mM$ and $c_{but}^{in}=6.25\times 10^{-6}\;mM$ .

The reference diet was the one used to simulate the healthy reference state (see §3.1), featuring a volume fraction of fibre entering the first section $f_{pol}^{in}=0.05$ and an entering protein fraction volume $f_{prot}^{in}=0.0135$ . For this diet, inputs were $c_{mon}^{in}=3.33\times 10^{-5}\;mM$ , $c_{la}^{in}=3\times 10^{-6}\;mM$ , $c_{ac}^{in}=6\times 10^{-5}\;mM$ , $c_{pro}^{in}=2\times 10^{-5}\;mM$ and $c_{but}^{in}=2\times 10^{-5}\;mM$ .

This comparison enabled us to evaluate how dietary habits, specifically the equilibrium between protein and fibre, could influence the resilience of a healthy host–microbiota symbiosis under stress conditions.

To carry out our investigation, we adopted a systematic approach to examine the effects of disturbances on our model. Initially, we exclusively explored the increase in AMP production rate (scenario A). Next, we added the influence of the oxygen bloom for a holistic study in scenario B. For both scenarios, considering their biological implications, we monitored the same outputs as the previous study: the oxygen level, the proportion of $B_{H_{2}s}^{\delta }$ in the lumen, and the mucus production rate, which depends on the density of differentiated cells and the activation of PRRs (electronic supplementary material, Eq. A.2). Specifically, for scenario B, we also evaluated the breach’s effect on cell densities. The case with only the effect of oxygen bloom is proposed as scenario C in electronic supplementary material, appendix D.

#### 3.3.3. Scenario’s results

##### 3.3.3.1. Scenario A: increased antimicrobial peptide production

**Figure 14.**
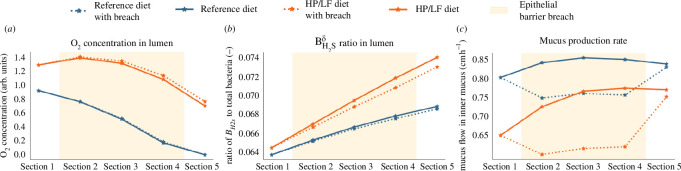
Scenario A: effects of increased AMP secretion. This figure showcases the effects of a surge in AMP secretion, simulating a reduction in mucus quality, under two different dietary regimes (reference and HP/LF diets).

The first scenario involved an increase in AMP production, representative of another potential breach in the epithelial barrier where mucus quality would have been reduced and the innate immunity reacted by increasing AMP production. This escalation did not significantly affect the oxygen concentration in the lumen but did contribute to a slight increase under the HP/LF diet regime (figure 14*a*).

Interestingly, one might have anticipated a higher ratio of $B_{H_{2}s}^{\delta }$ in the scenario with disturbance. However, our observations reveal the opposite, even if the disparity is minimal in both regimes. This counterintuitive outcome further underscores the complex interplay within the system. This decline can be traced back to $B_{H_{2}s}^{\delta }$ reduced presence in the outer mucus compartment, attributed to diminished mucoproteins. This effect in the outer mucus compartment then impacts the lumen owing to the movement of bacteria between these compartments (figure 14*b*).

With respect to mucus production, the rise in AMP production leads to a decrease, consistent with the reduction in the number of bacteria necessary for activating PRRs, in the mucus-producing goblet cells (figure 14*c*).

##### 3.3.3.2. Scenario B: combined oxygen bloom and increased antimicrobial peptide production

**Figure 15.**
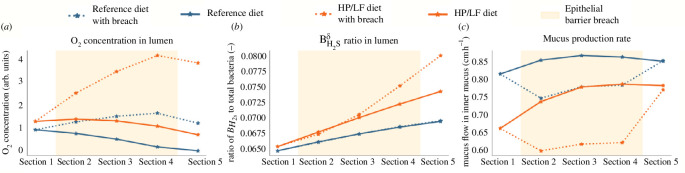
Scenario B: combined effects of an oxygen bloom and increased AMP secretion. This figure demonstrates the combined impact of an oxygen bloom at the crypt base and an increased AMP secretion, under two different dietary regimes (reference and HP/LF diets).

The second scenario combined the impacts of both an oxygen bloom at the crypt base and increased AMP production. Oxygen levels in the lumen increased, with a more exacerbate effect observed under the HP/LF dietary regime (figure 15*a*).

The behaviour of $B_{H_{2}s}^{\delta }$ bacteria in this scenario is twofold: up to section 2, we observe effects related to increased AMP production with a slight decrease in the ratio. Then, from section 3 onwards, we notice a surge in $B_{H_{2}s}^{\delta }$ bacteria (figure 15*b*). Finally, in terms of mucus production, the responses mirror those found in scenario A, where a higher AMP production leads to a decrease in mucus production (figure 15*c*).

##### 3.3.3.3. Effect on the crypt cell densities

In our study, we analysed the effect of diet and breach (scenario B) on cell densities, as presented in figure 13. One observation is the effect of diet on the crypt’s maturity. With the HP/LF diet, we identified an immature crypt profile characterized by a higher number of proliferative cells located higher up within the crypt combined with a lower number of differentiated cells. This implies that the HP/LF diet qualitatively encourages the formation of immature crypts in our context.

Moreover, our simulation indicates a tendency of cell crypts under HP/LF diet to use self-regulation to revert to a healthy state (figure 13*c*). This is evidenced by the progressive modulation of cell densities across sections; from section 1 to section 5, there is a decline in the number and prominence of proliferative cells within the crypt (from 852 in section 1 to 678 in section 5). Concurrently, the count of differentiated cells increases, moving from 1322 in section 1 to 1514 in section 5.

Our model predicts that while the dietary impact appears to be more pronounced on cell densities, the epithelial breach also plays a role across both diet types. Specifically, breaches visually amplify the presence of proliferative cells to the detriment of differentiated ones (figure 13*b*) and for the HP/LF diet, our simulation suggests that the breaches have a slightly more pronounced impact in proportion, delaying the self-regulation tendency observed in the absence of breach (figure 13*d*).

As a whole, our simulations indicate a higher sensitivity to inflammation and breaches for the HP/LF diet, which is consistent with the inflammation experiment in the context of the HP diet presented in [38].

## 4. Conclusive discussions

This study uses a comprehensive simulation model to explore nuanced interactions between the host and microbiota within the colon, discerning implications of dietary variations, particularly in the face of disturbances like epithelial breaches.

Built upon works by [20,21,25], our simulations, operating at a colon section scale, depicted epithelial crypt dynamics influenced by the colonic environment. Shaped by bacterial groups categorized by metabolic capabilities, our model introduced a bacterial group distribution correlating metabolism with inflammation sensitivity, enriching our representation of host–microbiota interactions by modelling innate immunity mechanisms at the epithelial scale.

We introduced new metabolic pathways, such as protein degradation, leading to $H_{2}S$ production, associated with a bacterial group gathering sulfate-reducing bacteria and cysteine catabolizers [31]. Additionally, by including oxygen diffusion, our model proposed a new interaction mechanism focusing on bacterial tolerance to oxygen in the environment, contributing to dysbiotic equilibrium onset.

Results highlight the intricate nature of the host–microbiota relationship, emphasizing vulnerability to dietary changes, especially HP/LF diets. Simulations underscored the positive influence of total nutrient intake on innate immunity receptors and the impact of protein/fibre ratios on facultative anaerobic bacterial groups. Fibre intake promoted crypt maturity, vital for absorption, leading to a hypoxic environment via differentiated cell oxygen consumption.

Findings accentuated the effects of diet on gut health, particularly under challenges like epithelial breaches. HP/LF diet amplified challenges, fostering conditions conducive to dysbiosis, encouraging facultative bacteria proliferation, escalating inflammation and pushing the microbiota towards a dysbiotic state. The HP/LF diet hindered mucus production, crucial for modulating host–microbiota interactions, suggesting diminished gut resilience under such diets. Our modelling approach is, therefore, consistent with the alternative stable state theory, suggesting a mechanistic view of the destabilizing effect of environmental factors such as diet and highlighting a possible transition to dysbiosis in the context of an imbalanced diet.

While our model provides valuable insights, it also highlights areas for improvement. These include expanding the scope of the protein degradation pathway, refining the characterization of the hydrogen sulfide-producing bacterial group, integrating representations of alternative bacterial metabolisms, and incorporating factors such as periodic variations in dietary supply and oxygen concentration. Additionally, considering variations in mucus layer thickness could offer a more holistic view of the system. In its current form, the model has a fixed input on $\Gamma^{in}$ which restricts our analysis to transitory disturbance within the sections. To address this situation, future iterations of the model could integrate the two-dimensional colon model from [20]. We acknowledge the model focuses on the epithelial cells’ innate immunity. Including more information on the role of dcs cells in the crypt and a lamina propia or a blood compartment with the presence of immune cells could be a more sophisticated depiction of the immune system. Moreover, introducing a bacterial virulence index might offer insights into targeted immune reactions.

In conclusion, this study represents a step forward in understanding the host–microbiota symbiosis within the colon. It showed the complex interplay between diet, health and the gut’s microbial environment. Despite areas for potential improvement, the current model provides critical insights into factors such as oxygen concentration, protein degradation, primary immunity mechanisms and the overall coupling effects within the colon ecosystem. The insights collected invite further exploration, particularly the potential for experimental validation. Such efforts could enrich our understanding of host–microbiota dynamics under various dietary conditions. Thus, our work lays a solid foundation for future exploration, investigation and refinement, deepening our understanding of the intricate dynamics of host–microbiota symbiosis and the influence of diet.

## Data Availability

Our open-source Python code is available from the Gitlab Repository: https://gitlab.com/marie.haghebaert/hostmicrobiota-interactions shared in Zenodo: [39] Supplementary material is available online at [40].
